# Skipping Breakfast and Eating Breakfast Away From Home Were Prospectively Associated With Emotional and Behavioral Problems in 115,217 Chinese Adolescents

**DOI:** 10.2188/jea.JE20210081

**Published:** 2022-12-05

**Authors:** Wei-Jie Gong, Daniel Yee-Tak Fong, Man-Ping Wang, Tai-Hing Lam, Thomas Wai-Hung Chung, Sai-Yin Ho

**Affiliations:** 1 School of Nursing, Li Ka Shing Faculty of Medicine, The University of Hong Kong, Hong Kong, China; 2 School of Public Health, Li Ka Shing Faculty of Medicine, The University of Hong Kong, Hong Kong, China; 3 Family & Student Health Branch, Department of Health, Hong Kong, China

**Keywords:** breakfast habit, emotional problems, behavioral problems, adolescent

## Abstract

**Background:**

Breakfast is deemed the most important meal of the day. We examined the prospective associations of breakfast habits with emotional/behavioral problems in adolescents and potential effect modification.

**Methods:**

115,217 Primary 6 students (United States Grade 6; mean age, 11.9; standard deviation [SD], 0.59 years) who attended the Student Health Service of Department of Health in Hong Kong in 2004/05, 2006/07, 2008/09 were followed till Secondary 6 (United States Grade 12). Emotional/behavioral problems were biennially examined using Youth Self-Report since Secondary 2 (United States Grade 8). Lifestyles were biennially examined using standardized questionnaires since Primary 6. Prospective associations of breakfast habit with emotional/behavioral problems and potential effect modification were examined using generalized estimating equations.

**Results:**

Compared with eating breakfast at home, eating breakfast away from home was significantly associated with total emotional/behavioral problems and seven syndromes, including withdrawal, somatic complaints, anxiety/depression, thought problems, attention problems, delinquent behaviors, and aggressive behaviors (adjusted odds ratios [AORs] 1.22–2.04), while skipping breakfast showed stronger associations with the above problems and social problems (AORs 1.34–2.29). Stronger associations were observed in younger students for total and attention problems (*P* < 0.03) and in those with lower weight status for delinquent behaviors (*P* = 0.005).

**Conclusion:**

Eating breakfast away from home and especially skipping breakfast were prospectively associated with adolescent emotional/behavioral problems. The associations weakened with increasing age for total emotional/behavioral and attention problems, and weakened with higher weight status for delinquent behaviors, highlighting the vulnerability of younger and underweight children. If the associations are causal, increasing home breakfast may reduce adolescent emotional/behavioral problems and benefit psychosocial health.

## INTRODUCTION

Adolescents are vulnerable to psychological problems with long-term implications on health and well-being.^1^ Half of all mental disorders are symptomatic by the age of 14, although most of them remain undiagnosed and untreated until later in life.^2^ Worldwide, around 10–20% of children and adolescents are affected by mental health problems.^3^ Moreover, adolescents with aggressive and delinquent behaviors have a high risk of becoming adult offenders.^4^ Such emotional and behavioral problems in adolescents could be influenced by their lifestyles and health-related behaviors,^5^ including physical activity and dietary habits.^6^^,^^7^

Breakfast has been widely regarded as the most important meal of the day, especially for children and adolescents,^8^^,^^9^ providing energy and nutrients for their growing body after fasting overnight.^10^ Regular breakfast consumption promotes youth’s nutritional status, weight maintenance,^11^^,^^12^ and cognitive performance.^8^^,^^13^ In contrast, poor breakfast habits, including skipping breakfast, may lead to the deficiency of many micronutrients that may not be compensated for by other meals.^14^^–^^16^ Poor breakfast habits can also cause metabolic inflexibility with higher postprandial insulin levels and increased fat oxidation, resulting in low-grade inflammation and impaired glucose homeostasis in the long run.^16^ Despite the importance of breakfast, 10–30% of adolescents from 33 countries and regions skipped breakfast, and the trend was increasing.^17^

Previous studies mostly focused on the benefits of breakfast on children’s mental health.^18^^,^^19^ Only several cross-sectional studies have linked breakfast skipping to emotional and behavioral problems, including depression, school bullying, and stress symptoms.^20^^–^^22^ No longitudinal evidence has been reported.

Also, with easy access to fast food, more children eat breakfast away from home.^23^^,^^24^ Foods served away from home are typically high-sugar products with lower nutrition quality,^25^ which have been linked to emotional symptoms in children.^26^ However, whether eating breakfast away from home is prospectively associated with emotional and behavioral health remains unknown. Whether there are interactions among these associations is also unclear. We hypothesized that breakfast location and breakfast skipping were associated with adolescent psychosocial health. Therefore, we aimed to assess the prospective associations of breakfast skipping and eating breakfast away from home with emotional and behavioral problems using longitudinal data of Chinese adolescents in Hong Kong and to explore potential effect modifiers.

## METHODS

### Design and subjects

This was a territory-wide population-based longitudinal study. All subjects were participants of the Student Health Service (SHS) of the Department of Health, Hong Kong Special Administrative Region, China. The SHS provided free voluntary annual health assessments for all primary and secondary school students in 12 SHS centers, which covered all districts in Hong Kong. Students were invited to complete a standardized questionnaire on lifestyles in Primary 4 (P4 or United States Grade 4), P6, Secondary 2 (S2 or United States Grade 8), S4, and S6. They also completed the Youth Self-Report (YSR) for assessing emotional and behavioral problems in S2, S4, and S6. Most (97%) students completed the Chinese version of YSR while the English version was also available. We obtained anonymous data from the SHS, with longitudinal data for each student linked using a unique identification number.

P6 students in the academic years of 2004/05, 2006/07, and 2008/09 were enrolled in this study and were tracked for 6 years until S6 in 2010/11, 2012/13, and 2014/15, respectively. P6 students in 2005/06 and 2007/08 were not included because they could not attend the SHS appointments in their S4 and S2, respectively, in 2009/10 when the SHS was engaged in the Human Swine Influenza Vaccination Programme. A total of 175,113 P6 students were included, corresponding to 74.8% of all P6 students in Hong Kong in the academic years of 2004/05, 2006/07, and 2008/09.^27^

The participants’ parents/guardians gave written consent each year to enroll in the annual SHS health assessments, including use of the participants’ data for research.^28^ The study protocol was approved by the Institutional Review Board of The University of Hong Kong/Hospital Authority Hong Kong West Cluster (Reference number: UW 19-206) and the Department of Health Ethics Committees (Reference number: L/M 66/2019).

### Measurements

The outcomes were emotional/behavioral problems in S2, S4, and S6 using YSR, which was shown to be reliable and valid in Hong Kong children and adolescents.^29^^,^^30^ YSR consists of 112 items with a 3-point response scale (scores): not true (0), somewhat or sometimes true (1), and very true or often true (2).^31^ It has a total scale and eight syndrome subscales, with scores above the local threshold indicating the respective problems. A total score of over 71 in boys and 78 in girls indicates total emotional and behavioral problems. Threshold scores for the subscales (boys, girls) are: withdrawal (9, 10), somatic complaints (9, 10), anxiety/depression (19, 21), social problems (10, 10), thought problems (8, 8), attention problems (13, 13), delinquent behaviors (9, 8) and aggressive behaviors (21, 20).^29^ The thresholds were developed using local Hong Kong norms.^32^

Lifestyles were assessed in P6, S2, and S4 using a set of closed-ended items. The study factor of breakfast habit was assessed by the item ‘I usually have breakfast at…’ with four response options: (i) ‘no breakfast at all’ denoted skipping breakfast, (ii) ‘home’ denoted eating at home, (iii) ‘fast-food stall/cafeteria/restaurant’ and (iv) ‘some other places’ denoted eating breakfast away from home. The frequency and duration of extracurricular aerobic physical activities, such as ball sports, swimming, and running, were dichotomized as <1 or ≥1 time/week and <60 or ≥60 minutes/week, respectively.

Sex, age, weight, height, and socioeconomic status were also obtained from the SHS. Weight (to the nearest 0.1 kg) and height (to the nearest 0.1 cm) were annually measured by well-trained healthcare staff following standard procedures. Socioeconomic status included parental educational level (primary or below, secondary, and tertiary) and parental occupation (unemployed, manual job, clerical/service industry, and managerial/professional job).

### Statistical analysis

Body mass index (BMI) was calculated as weight (kg) divided by squared height (m^2^). According to the International Obesity Task Force standards, weight status was classified as underweight, normal, overweight and obese using age-sex-specific BMI cut-off values that correspond to BMI values of 18.5, 25.0, and 30.0 kg/m^2^ at 18 years of age.^33^ To examine whether the included sample was representative, the characteristics of the participants were compared with those of the corresponding children and adolescent population in Hong Kong^34^ using Cohen’s d for age, with values of 0.20, 0.50, and 0.80 denoting small, medium, and large effect sizes, respectively,^35^ and Cramer’s V for categorical variables, with values of 0.10, 0.30, and 0.50 denoting small, medium, and large effect sizes, respectively.^36^

The characteristics of adolescents were compared among those eating breakfast at home, away from home, and skipping breakfast in P6 using the Kruskal-Wallis test and Chi-square test for continuous and categorical characteristics, respectively. The prospective associations of breakfast habit (eating breakfast at home, away from home, and skipping breakfast) with the outcomes were examined using generalized estimating equations (GEE) models with a logit link. Specifically, the main exposure was breakfast habit, which was time-dependent and measured in P6, S2, and S4, and the outcomes were the presence of total emotional/behavioral problems and the eight syndromes, which were measured in S2, S4, and S6. Unstructured correlation matrix was used to account for the extra-covariance among the longitudinal measurements. An adjustment was made for sex, parental educational level and parental occupation, year enrolled in P6, and time-dependent factors, including age, weight status, and frequency/duration of extracurricular physical activity in P6, S2, and S4. The Benjamini-Hochberg correction was used to account for multiple testing of different emotional and behavioral problems,^37^ while the Dunnett-Hsu method was used to adjust the *P*-values of multiple pairwise comparisons across breakfast habits. Adjusted odds ratios (AORs) with 95% confidence intervals (CIs) were reported. Tests for trend across breakfast habits were performed by including breakfast habits as a three-category ordinal variable (eating breakfast at home, away from home, and skipping breakfast as 1, 2, and 3, respectively) in the respective GEE models.^38^ The moderating effects of sex, age, weight status, parental educational level, and parental occupation with breakfast habits were estimated on all the outcomes by incorporating the corresponding interaction terms. Statistically significant interactions were estimated using the ratio of odds ratios (RORs). A ROR greater than 1 indicates that the differences in the odds ratios increased between the compared breakfast habits. For statistically significant interactions, the predicted probabilities of outcomes across breakfast habits were estimated in the respective GEE models and plotted in separate lines across the moderators. To examine the slope of separate lines of breakfast habits, tests for trend were used by including the moderators as continuous variables in the respective GEE models.^38^

In addition, we estimated the mean and standard error of the total and subscale YSR scores across sex, grade, and breakfast habits, and their adjusted regression coefficients (βs) with 95% CIs using GEE models, adjusted for academic year enrolled in P6, parental educational level and occupation, and time-dependent factors in Primary 6 and Secondary 2 and 4, including weight status and frequency/duration of extracurricular physical activity, and mutually adjusted for each other. All analyses were conducted using the Statistical Analysis System (SAS Institute, Cary, NC, USA) 9.4 with a two-tailed significance level of 0.05.

## RESULTS

Among the 175,113 P6 students who attended the SHS service, 55,117 (31.5%) with no records in S2, S4, or S6; 1,972 (1.1%) with no breakfast habit data in P6; and 2,807 (1.6%) with missing data of socioeconomics and/or frequency/duration of extracurricular physical activity in P6 were excluded. After removing them from the present analysis, 115,217 (65.8%) students (girls 52.2%; mean age 11.9; standard deviation [SD], 0.59 years) remained, with an average of 1.7 (SD, 0.73) follow-up records (range, 1 to 3). The differences of the characteristics between the included sample and the corresponding Hong Kong population were small, with all Cramer’s V ≤0.10 (eTable 1). The differences of the P6 characteristics between the included and excluded samples were also small, with Cohen’s d = 0.16 for age and all Cramer’s V for categorical characteristics ≤0.10 (eTable 2).

In P6, 84.5%, 8.7%, and 6.8% of participants ate breakfast at home, away from home, and skipped it, respectively. Table 1 shows significant differences in sex, weight status, parental educational level, parental occupation, frequency and duration of physical activity, and year enrolled in P6. Compared with children who ate breakfast at home, those who ate breakfast away from home or skipped breakfast had a higher percentage of being overweight (19.7% and 22.2% vs 15.8%) and obese (4.6% and 5.4% vs 2.8%), and a lower percentage of having tertiary-educated parents (11.4% and 10.6% vs 17.2%), parents working in managerial/professional position (18.6% and 18.6% vs 24.3%), and doing physical activity ≥1 time/week (74.2% and 66.1% vs 76.7%) (all *P* < 0.001).

**Table 1.** Baseline characteristics of students by breakfast habits in Primary 6, *n* (%)

Characteristics	All (*n* = 115,217)	Breakfast habits			*P* ^a^

		Eating at home (*n* = 97,405)	Eating away from home (*n* = 9,994)	Skipping (*n* = 7,818)	
Age, mean (SD), years	11.9 (0.59)	11.9 (0.59)	12.0 (0.59)	12.0 (0.65)	<0.001
Sex					<0.001
Male	55,027 (47.8)	46,171 (47.4)	5,171 (51.7)	3,685 (47.1)	
Female	60,190 (52.2)	51,234 (52.6)	4,823 (48.3)	4,133 (52.9)	
Weight status					<0.001
Underweight	16,208 (14.1)	14,238 (14.6)	1,184 (11.9)	786 (10.1)	
Normal	76,351 (66.3)	65,099 (66.8)	6,380 (63.8)	4,872 (62.3)	
Overweight	19,041 (16.5)	15,337 (15.8)	1,969 (19.7)	1,735 (22.2)	
Obese	3,617 (3.1)	2,731 (2.8)	461 (4.6)	425 (5.4)	
Parental educational level					<0.001
Primary or below	13,953 (12.1)	11,595 (11.9)	1,286 (12.9)	1,072 (13.7)	
Secondary	82,546 (71.6)	69,060 (70.9)	7,569 (75.7)	5,917 (75.7)	
Tertiary	18,718 (16.3)	16,750 (17.2)	1,139 (11.4)	829 (10.6)	
Parental occupation					<0.001
Unemployed	6,110 (5.3)	5,069 (5.2)	527 (5.3)	514 (6.6)	
Manual job	39,392 (34.2)	33,340 (34.2)	3,447 (34.5)	2,605 (33.3)	
Clerical/service industry	42,764 (37.1)	35,354 (36.3)	4,162 (41.6)	3,248 (41.6)	
Managerial/professional	26,951 (23.4)	23,642 (24.3)	1,858 (18.6)	1,451 (18.6)	
Frequency of extracurricular physical activity					<0.001
<1 time/week	27,962 (24.3)	25,736 (23.3)	2,573 (25.8)	2,653 (33.9)	
≥1 time/week	87,255 (75.7)	74,669 (76.7)	7,421 (74.2)	5,165 (66.1)	
Duration of extracurricular physical activity					<0.001
<60 minutes/week	71,842 (62.4)	60,515 (62.1)	6,188 (61.9)	5,139 (65.7)	
≥60 minutes/week	43,375 (37.7)	36,890 (37.9)	3,806 (38.1)	2,679 (34.3)	
Year in Primary 6					
2004/05	39,230 (34.1)	33,435 (34.3)	3,267 (32.7)	2,528 (32.3)	<0.001
2006/07	40,934 (35.5)	34,543 (35.5)	3,494 (35.0)	2,897 (37.1)	
2008/09	35,053 (30.4)	29,427 (30.2)	3,233 (32.4)	2,393 (30.6)	

SD, standard deviation.

^a^Based on Kruskal-Wallis test for age, and Chi-square test for other variables.

Table 2 shows that, compared with students who ate breakfast at home, those who ate away from home had significantly higher AORs of total emotional/behavioral problems (AOR 1.45) and seven syndromes, including withdrawal (AOR 1.22), somatic complaints (AOR 1.40), anxiety/depression (AOR 1.42), thought problems (AOR 1.27), attention problems (AOR 1.52), delinquent behaviors (AOR 2.04) and aggressive behaviors (AOR 1.55) (*P* = 0.016 to <0.001) but not social problems (AOR 1.14; 95% CI, 0.93–1.39, *P* = 0.21). Skipping breakfast was associated with higher AORs of total emotional/behavioral problems (AOR 1.87) and all eight syndromes (AORs 1.34 to 2.29) (*P* = 0.008 to <0.01). Compared with students who ate breakfast away from home, those who skipped breakfast had higher AORs of total emotional/behavioral problems (AOR 1.28) and three syndromes including somatic complaints (AOR 1.51), thought problems (AOR 1.31), and aggressive behaviors (AOR 1.34) (*P* = 0.04 to <0.001).

**Table 2.** The prospective associations of breakfast habits in Primary 6, Secondary 2 and Secondary 4 with emotional and behavioral problems in Secondary 2, 4, and 6^a^

Emotional/behavioral problems	*P* for overall association^b^	Eating away from home vs Eating at home	Skipping vs Eating at home	*P* for trend	Skipping vs Eating away from home
The total emotional/behavioral problems	<0.001	**1.45 (1.30, 1.62)** ^e^	**1.87 (1.66, 2.10)** ^e^	<0.001	**1.28 (1.11, 1.48)** ^d^
Withdrawal	<0.001	**1.22 (1.04, 1.44)** ^c^	**1.52 (1.27, 1.82)** ^e^	<0.001	1.24 (1.00, 1.55)
Somatic complaints	<0.001	**1.40 (1.19, 1.65)** ^e^	**2.12 (1.81, 2.48)** ^e^	<0.001	**1.51 (1.23, 1.85)** ^e^
Anxiety/depression	<0.001	**1.42 (1.18, 1.70)** ^e^	**1.70 (1.40, 2.06)** ^e^	<0.001	1.19 (0.94, 1.53)
Social problems	<0.001	1.14 (0.93, 1.39)	**1.34 (1.08, 1.66)** ^d^	0.009	1.18 (0.89, 1.54)
Thought problems	<0.001	**1.27 (1.05, 1.54)** ^c^	**1.67 (1.37, 2.04)** ^e^	<0.001	**1.31 (1.02, 1.70)** ^c^
Attention problems	<0.001	**1.52 (1.28, 1.80)** ^e^	**1.82 (1.51, 2.19)** ^e^	<0.001	1.19 (0.95, 1.50)
Delinquent behaviors	<0.001	**2.04 (1.71, 2.44)** ^e^	**2.29 (1.89, 2.79)** ^e^	<0.001	1.12 (0.89, 1.42)
Aggressive behaviors	<0.001	**1.55 (1.29, 1.85)** ^e^	**2.08 (1.73, 2.49)** ^e^	<0.001	**1.34 (1.06, 1.70)** ^c^

^a^Adjusted odds ratios (95% confidence interval) with eating breakfast at home or eating breakfast away from home as the reference group, adjusted for sex, parental educational level and occupation status, enrolled year in Primary 6 and age, weight status and frequency/duration of extracurricular physical activity in Primary 6, Secondary 2 and 4.

^b^The overall *P*-values tested about the null hypothesis of no difference in the odds of an emotional/behavioral problem across all breakfast habits. *P*-values were adjusted by the Benjamini-Hochberg correction.

^c^*P* < 0.05.

^d^*P* < 0.01.

^e^*P* < 0.001.

**Figure 1.**
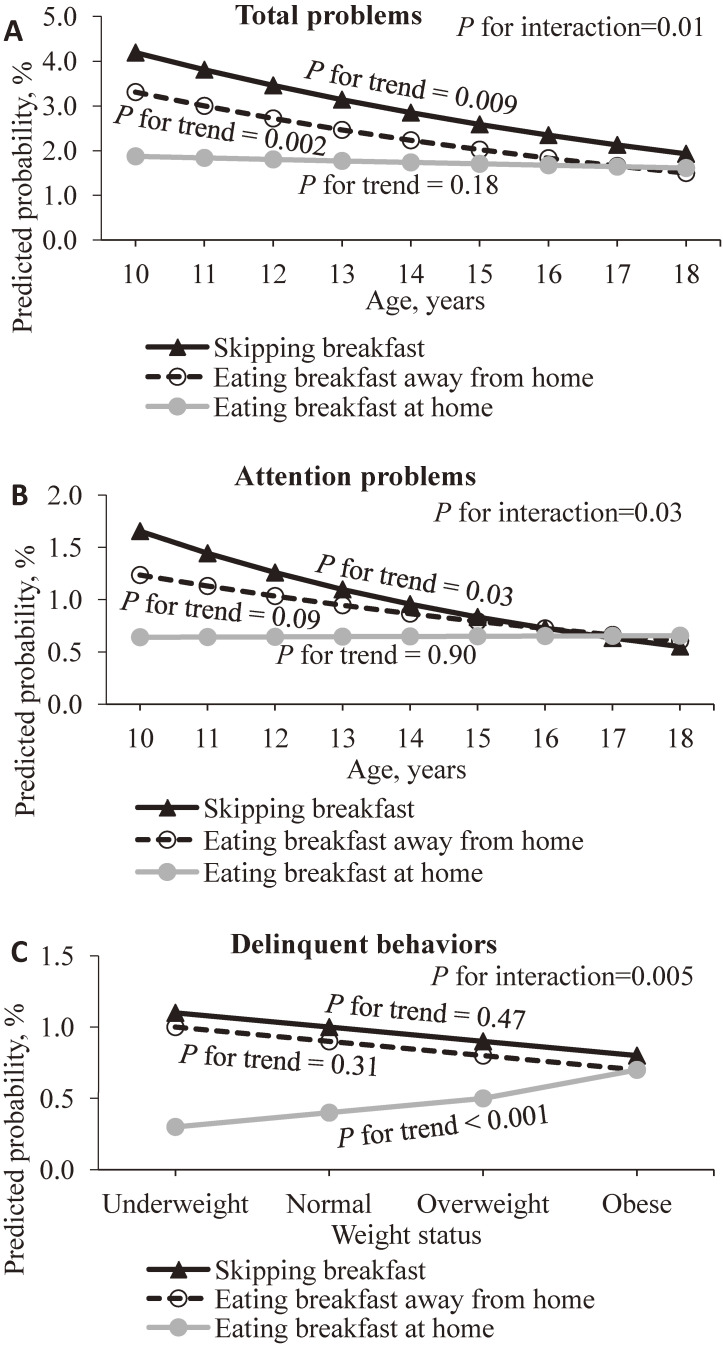
The predicted probability (%) of (**A**) the total emotional/behavioral problems and (**B**) attention problems by breakfast habits and age, as well as (**C**) delinquent behaviors by breakfast habits and weight status.^a^ ^a^All predicted probabilities were estimated using generalized estimating equations with a logit link, with having the corresponding problems as 1, adjusted for sex, parental educational level and parental occupation, year enrolled in P6 and time-dependent factors, including age, weight status and frequency/duration of extracurricular physical activity.

Only three interactions were statistically significant (Figure 1), including those between age and breakfast habit for total emotional/behavioral problems (*P* = 0.01) and attention problems (*P* = 0.03), as well as that between weight status and breakfast habit for delinquent behaviors (*P* = 0.005). Figure 1A shows that as students grew from 10 to 18 years of age, the predicted probabilities of total emotional/behavioral problems were stable for those who ate breakfast at home (*P for trend* = 0.18) but decreased for those who ate breakfast away from home (*P for trend* = 0.002) or skipped breakfast (*P for trend* = 0.009). In general, the predicted probabilities of total emotional/behavioral problems for eating breakfast at home were lower than those for eating breakfast away from home or skipping breakfast. However, the differences decreased (eating breakfast away from home: ROR 0.92; 95% CI, 0.86–0.99, *P* = 0.04 and skipping breakfast: ROR 0.92; 95% CI, 0.85–1.00, *P* = 0.02) with increasing age. Figure 1B shows similar trends in the predicted probabilities of attention problems. Similarly, the predicted probabilities of attention problems for eating breakfast at home was lower than those for eating breakfast away from home or skipping breakfast. The difference with skipping breakfast decreased significantly (ROR 0.87; 95% CI, 0.74–0.99, *P* = 0.03) but that with eating breakfast away from home was not significant (ROR 0.91; 95% CI, 0.82–1.02, *P* = 0.11). Figure 1C shows that with higher weight status, the predicted probabilities of delinquent behaviors increased for eating breakfast at home (*P* for trend <0.001) but was stable for eating breakfast away from home (*P for trend* = 0.31) and skipping breakfast (*P for trend* = 0.47). The predicted probabilities of delinquent behaviors for eating breakfast at home was generally lower than those for eating breakfast away from home or skipping breakfast. However, the difference decreased (eating breakfast away from home: ROR 0.69; 95% CI, 0.53–0.90, *P* < 0.01 and skipping breakfast: ROR 0.72; 95% CI, 0.55–0.93, *P* = 0.01) with higher weight status.

When taking the total and subscale YSR scores as continuous outcomes, as shown in eTable 3, girls had higher scores than boys for total emotional/behavioral problems and all subscale problems except for lower scores of delinquent behaviors (all *P* ≤ 0.04). Participants in higher grades had higher scores in total scores and the scores of withdrawal, somatic complaints, anxiety/depression, attention problems, and delinquent problems (all *P* < 0.001). Compared with eating breakfast at home, eating breakfast away from home and skipping breakfast predicted higher scores of total problems (adjusted βs: 2.60 and 5.99, respectively) and all syndrome scores (adjusted βs: 0.07–0.66 and 0.31–1.21, respectively) (all *P* < 0.001).

## DISCUSSION

This is the first longitudinal study showing that both eating breakfast away from home and skipping breakfast were associated with emotional and behavioral problems in adolescents. Eating breakfast away from home was associated with higher odds of total emotional/behavioral problems and seven syndromes except for social problems, and skipping breakfast was associated with higher odds of total emotional/behavioral problems and all eight syndromes. Age modified the associations of breakfast habits with the total emotional/behavioral problems and attention problems, with stronger associations at younger ages. Weight status modified the association between breakfast habits and delinquent behaviors, with the predicted probabilities of delinquent behaviors slightly increased with higher weight status only for eating breakfast at home but was stable for eating breakfast away from home or skipping breakfast.

Among the three breakfast habits, eating breakfast at home was the most beneficial one for adolescent psychosocial health. Compared with eating breakfast at home, eating breakfast away from home predicted higher odds of total emotional/behavioral problems and several syndromes. Considering that home meals were reported to have superior nutritional quality to those served away from home in Western countries,^25^^,^^39^ breakfast nutrition could be one possible reason, but this may differ across cultures, which warrants detailed dietary assessment of local breakfast, including portion size, food variety, and nutrition quality. Meanwhile, even for the same food, eating at home may result in better emotional reinforcement than eating away from home.^39^ Home meals in post-meal affective states were found to have greater emotional reinforcing values than meals away from home.^3^ This may start from the parent-child attachment relationship since the early stage of life. When trusting and caring parents provide food to their children, the secure home environment could help establish a secure attachment,^39^ which is important to the development of children’s psychosocial health. Besides, adolescents who ate away from home or skipped breakfast might have parents who are busier or less caring, with less involvement in preparing breakfast meals and therefore have less parental monitoring and family communication, resulting in higher risks of emotional and behavioral problems.

Compared with eating breakfast away from home, skipping breakfast was prospectively associated with total emotional/behavioral problems and several syndromes. There are several possible reasons. Mood regulation is a complex result of a series of different neurochemical pathways, each requiring several nutrients to supply the necessary metabolites, such as serotonin and dopamine.^40^^,^^41^ Nutrition supplementations in school children, including multivitamins, minerals, and *n*-3 fatty acids, have shown to be effective in treating depression and attention deficits^42^^–^^44^ and reducing institutional violence and antisocial behaviors.^45^ Compared with children eating breakfast away from home, habitual breakfast skippers were more likely to have a deficiency in these essential micronutrients that produce the involved neurotransmitters.^14^^–^^16^ These micronutrient deficiencies could cause impaired brain function, attention deficits, and poorer cognitive performance,^46^ which subsequently lead to different syndromes of emotional and behavioral problems. Also, low frequency of eating breakfast was associated with low family functioning, including worse relations with parents, lower quality of family communication, and less family support.^47^ Furthermore, breakfast skippers tend to have other health-compromising lifestyles, including sedentary behaviors, smoking, and other unhealthy dietary habits,^48^^,^^49^ which may have combined effects on adolescent emotional and behavioral problems. More evidence is needed to unravel the underlying mechanisms.

We have also reported, for the first time, the interaction between breakfast habits with weight status on delinquent behaviors (Figure 1C). With higher weight status, eating breakfast away from home and skipping breakfast showed stable predicted probabilities of delinquent behaviors (*P* for trend ≥0.31), whereas eating breakfast at home showed increasing predicted probabilities (*P* for trend <0.001). Insufficient nutrition intake from breakfast may be one of possible reasons for the adverse effects from unhealthy breakfast habits.^25^^,^^39^ However, as overweight and obese children tend to be over-nourished, they would be less susceptible to inadequate nutritional intake due to eating breakfast away from home or skipping breakfast. Therefore, their risks of delinquent behaviors showed smaller changes when they did not eat breakfast at home. Breakfast prepared at home may have higher nutrition quality, which would have more benefits to children at a lower weight status. In other words, eating breakfast at home had minimal, if any, beneficial effects in obese children for reducing their risks of developing delinquent behaviors. Further prospective or qualitative evidence considering other related factors, such as weight satisfaction and peer relationship, would be valuable.

Notably, we also found weaker associations of breakfast habits with total emotional/behavioral problems and attention problems with increasing age, indicating the vulnerability of younger children in psychosocial health when having unfavorable breakfast habits. Younger children are still in the early stage of physical and psychosocial development, so they may be more sensitive to the influence of breakfast habit. Special attention should be given to younger children to develop healthy dietary habits, although skipping breakfast was less prevalent in them.^50^ There have been tremendous increases in the consumption of food away from home and skipping breakfast globally.^23^^,^^24^^,^^50^ To promote these children to eat breakfast at home, the health awareness of their parents and families should be improved.

According to the guide that an OR of 2.0 is recommended as a ‘practically’ significant effect,^51^ the associations of eating breakfast away from home with delinquent behaviors, and the associations of skipping breakfast with somatic complaints, delinquent behaviors, and aggressive behaviors remain ‘practically’ significant, while the associations with withdrawal and social problems were relatively small. Our findings suggest that the associations of unhealthy breakfast habits may manifest more in adolescent externalizing behaviors (corresponding to delinquent and aggressive behaviors in YSR),^29^ such as lying/cheating, stealing, running away, arguing, fighting, and attacking, rather than inner distress. These findings support previous studies linking unhealthy diets with externalizing behaviors in children.^52^^,^^53^ We have first shown the associations of breakfast habits with somatic complaints in adolescents. Somatic complaints, withdrawal, and anxiety/depression comprised internalizing problems in YSR.^29^ Somatic complaints, including feeling dizzy or tired, having headaches, and vomiting, were found to vary by culture, with somatic expressions of distress more common in Asian people and affective expressions of distress more common in Western people.^54^^,^^55^ Further studies are warranted to explore the underlying mechanisms. Moreover, this study benefited from the large sample size to have statistically significant findings from associations with small effect size, which, however, should not be neglected due to the clustering of unhealthy behaviors. For example, insufficient sleep, pathological internet use, and online risky behaviors were found to be common and associated with breakfast skipping in Asian children,^56^^,^^57^ and they were all found to link with psychosocial problems.^58^^,^^59^ More evidence is needed on whether such cluster may have cumulative adverse effects on mental health in young people.

This study had some limitations. First, self-reported questionnaires may be subject to recall bias and social expectations bias. However, they are most viable in large-scale studies with a long follow-up. Second, as nutrition quality is often lower in breakfast served away from home than that in home-made breakfast, the behavioral pattern of purchasing breakfast away from home but eating it at home should be considered in future studies, which can examine the influences of home environment. Third, although school breakfast programs are widely provided in Western countries, school breakfast could not be included in this study due to the lack of such programs in Hong Kong. Fourth, despite our efforts to minimize residual confounding, there might remain other confounding factors relating to both breakfast habits and emotional/behavioral problems. For example, breakfast skipping may be associated with other unhealthy lifestyles, such as insufficient sleep and sedentary behaviors,^56^^,^^57^ and unfavorable family environment, such as poor family relationship and non-intact family. Therefore, only promoting eating breakfast at home could not solve all adolescent emotional/behavioral problems. Further studies are warranted to find effective psychosocial health promotion measures in children and adolescents. Lastly, there were noticeable missing values in this large-scale territory-wide study, and the attendance rate in follow-ups decreased with higher academic years. However, the small differences between the characteristics of our sample with the census data of the corresponding Hong Kong population indicated good representativeness (eTable 1), and only small effect sizes were found for the differences in baseline characteristics between the students who were and were not included in this study (eTable 2).

### Conclusion

Eating breakfast away from home and especially skipping breakfast were prospectively associated with more adolescent emotional/behavioral problems. The associations weakened with increasing age for total and attention problems, and weakened with higher weight status for delinquent behaviors, highlighting the vulnerability of younger and underweight children. If the associations are causal, increasing home breakfast may reduce adolescent emotional/behavioral problems and benefit psychosocial health.

## SUPPLEMENTARY MATERIAL

eTable 1. Comparison of baseline characteristics with a corresponding Hong Kong population

eTable 2. Baseline characteristics of students included or excluded in this study

eTable 3. Associations of sex, grade and breakfast habits with Youth Self-Report scores^a^
