# Supplementary material for: Skipping Breakfast and Eating Breakfast Away From Home Were Prospectively Associated With Emotional and Behavioral Problems in 115,217 Chinese Adolescents
Source: J Epidemiol. 2022 Dec 5;32(12):551–8. doi: 10.2188/jea.JE20210081 (PMC9643789; doi:10.2188/jea.JE20210081)
Supplement: Supplementary file 1 [file je-32-551-s001.pdf]

eTable 1. Comparison of baseline characteristics with a corresponding Hong Kong population

Characteristics	Corresponding Hong Kong population, *n* (%)[a]	Included sample, *n* (%)	Effect size[b]
Sex			0.03
Male	209,385 (51.3)	55,027 (47.8)	
Female	198,782 (48.7)	60,190 (52.2)	
Place of birth			0.02
Hong Kong	332,861 (80.4)	94,221 (81.8)	
Macau	1,249 (0.3)	256 (0.2)	
China	64,645 (15.6)	17,511 (15.2)	
Others	15,100 (3.6)	3,229 (2.8)	
Housing type		Missing = 1,532	0.003
Permanent housing	149,137 (36.0)	41,293 (36.3)	
Subsidized sale flats	68,427 (16.5)	18,693 (16.4)	
Private housing	191,745 (46.3)	52,462 (46.2)	
Non-domestic housing	4,413 (1.1)	1,237 (1.1)	
Residential district		Missing = 37	0.06
Hong Kong Island	66,971 (16.2)	20,542 (17.8)	
Kowloon	112,652 (27.2)	37,288 (32.4)	
New Territories	234,099 (56.6)	57,350 (49.8)	

[a] All comparison data of the corresponding Hong Kong population (aged 10–14 years) was from the Hong Kong 2006 Population By-census Report, including sex from the "Usual Residents by Quinquennial Age Group and Sex, 2006", place of birth from "Hong Kong Resident Population by Duration of Residence in Hong Kong, Marital Status, Place of Birth, Quinquennial Age Group and Sex, 2006", housing type from "Hong Kong Resident Population (Land) by Quinquennial Age Group, Sex and Type of Quarters, 2006", and residential district from "Hong Kong Resident Population by Quinquennial Age Group, Sex and Broad Area, 2006", and residential district from Cramer's V for categorical variables, with small, medium, and large effect sizes being defined by taking values of 0.10, 0.30 and 0.50, respectively.

[b] Cramer's V for categorical variables, with small, medium, and large effect sizes being defined by taking values of 0.10, 0.30 and 0.50, respectively.

eTable 2. Baseline characteristics of students included or excluded in this study

Characteristics	Excluded[a] (*n* = 59,896)	Included (*n* = 115,217)	Effect size[b]
Age, years, mean (SD)	12.0 (0.72)	11.9 (0.59)	0.16
Sex			0.08
Male	26,579 (44.4)	55,027 (47.8)	
Female	33,317 (55.6)	60,190 (52.2)	
Weight status			0.06
Underweight	7,171 (12.0)	16,208 (14.1)	
Normal	38,408 (64.2)	76,351 (66.3)	
Overweight	11,179 (18.7)	19,041 (16.5)	
Obese	3,074 (5.1)	3,617 (3.1)	
Parental educational level			0.05
Primary or below	8,681 (15.5)	13,953 (12.1)	
Secondary	39,453 (70.2)	82,546 (71.6)	
Tertiary	8,055 (14.3)	18,718 (16.3)	
Parental occupation			0.04
Unemployed	4,252 (7.5)	6,110 (5.3)	
Manual job	20,906 (35.6)	39,392 (34.2)	
Clerical/service industry	20,957 (35.6)	42,764 (37.1)	
Managerial/professional	12,690 (21.6)	26,951 (23.4)	
Breakfast habits			0.10
Skipping	6,650 (11.5)	7,818 (6.8)	
Eating away from home	6,619 (11.4)	9,994 (8.7)	
Eating at home	44,652 (77.1)	97,405 (84.5)	
Frequency of extracurricular physical activity			0.01
<1 time/week	13,237 (23.4)	27,962 (24.3)	
≥1 time/week	43,371 (76.6)	87,255 (75.7)	
Duration of extracurricular physical activity			0.03
<60 minutes/week	33,546 (59.6)	71,842 (62.4)	
≥60 minutes/week	22,727 (40.4)	43,375 (37.7)	

SD, standard deviation.

[a] 349 students had missing values in the total emotional/behavioral problems but data of other problems were included in this study.

[b] Cohen's d for age, with small, medium, and large effect sizes being defined by taking values of 0.20, 0.50 and 0.80, respectively. Cramer's V for categorical variables, with small, medium, and large effect sizes being defined by taking values of 0.10, 0.30 and 0.50, respectively.

eTable 3. Associations of sex, grade and breakfast habits with Youth Self-Report scores[a]

Characteristics	The total emotional/ behavioral problems		Withdrawal		Somatic complaints		Anxiety/ depression		Social problems	
	Mean (SE)	Adjusted β (95% CI)	Mean (SE)	Adjusted β (95% CI)	Mean (SE)	Adjusted β (95% CI)	Mean (SE)	Adjusted β (95% CI)	Mean (SE)	Adjusted β (95% CI)
Sex										
Male	30.30 (0.13)	1	2.96 (0.02)	1	1.81 (0.01)	1	4.51 (0.03)	1	2.86 (0.02)	1
Female	32.73 (0.13)	2.43 (2.23, 2.63)[d]	2.99 (0.02)	0.03 (0.01, 0.05)[c]	2.35 (0.02)	0.54 (0.52, 0.56)[d]	5.10 (0.03)	0.60 (0.55, 0.65)[d]	3.08 (0.02)	0.22 (0.20, 0.24)[d]
Overall *P*		<0.001		0.009		<0.001		<0.001		<0.001
Grade										
Secondary 2	30.60 (0.12)	1	2.70 (0.01)	1	1.88 (0.01)	1	4.52 (0.03)	1	3.01 (0.01)	1
Secondary 4	31.78 (0.13)	1.18 (1.05, 1.30)[d]	3.01 (0.01)	0.30 (0.28, 0.32)[d]	2.09 (0.01)	0.20 (0.19, 0.22)[d]	4.89 (0.03)	0.37 (0.34, 0.40)[d]	2.96 (0.01)	-0.05 (-0.07, -0.04)[d]
Secondary 6	32.15 (0.15)	1.55 (1.35, 1.75)[d]	3.23 (0.02)	0.52 (0.49, 0.55)[d]	2.26 (0.02)	0.37 (0.35, 0.40)[d]	5.00 (0.04)	0.48 (0.43, 0.53)[d]	2.93 (0.02)	-0.08 (-0.10, -0.05)[d]
Overall *P*		<0.001		<0.001		<0.001		<0.001		<0.001
Breakfast habits										
Eating at home	28.65 (0.12)	1	2.82 (0.01)	1	1.83 (0.01)	1	4.37 (0.03)	1	2.83 (0.01)	1
Eating away from home	31.25 (0.15)	2.60 (2.36, 2.83)[d]	2.89 (0.02)	0.07 (0.04, 0.10)[d]	2.08 (0.02)	0.25 (0.22, 0.28)[d]	4.67 (0.04)	0.30 (0.24, 0.35)[d]	2.94 (0.02)	0.11 (0.08, 0.13)[d]
Skipping	34.64 (0.18)	5.99 (5.69, 6.29)[d]	3.23 (0.02)	0.41 (0.38, 0.45)[d]	2.32 (0.02)	0.49 (0.45, 0.52)[d]	5.37 (0.04)	1.00 (0.93, 1.07)[d]	3.14 (0.02)	0.31 (0.28, 0.34)[d]
Overall *P*		<0.001		<0.001		<0.001		<0.001		<0.001

CI, confidence interval; SE, standard error.

[a] Higher scores indicate the presence of more symptoms of the corresponding problems. Mean (SE and adjusted regression coefficients (βs) (95% CIs) were estimated using generalized estimating equations with the Youth Self-Report scores as continuous outcomes, adjusted for year enrolled in P6, parental educational level and occupation, time-dependent factors in Primary 6 and Secondary 2 and 4, including weight status, and frequency/duration of extracurricular physical activity, and mutually adjusted for each other.

[b] $P<0.05$

[c] $P<0.01$

[d] $P<0.001$

eTable 3. Associations of sex, grade and breakfast habits with Youth Self-Report scores[a] (continued)

Characteristics	Thought problems		Attention problems		Delinquent behaviors		Aggressive behaviors	
	Mean (SE)	Adjusted β (95% CI)	Mean (SE)	Adjusted β (95% CI)	Mean (SE)	Adjusted β (95% CI)	Mean (SE)	Adjusted β (95% CI)
Sex								
Male	1.21 (0.01)	1	4.23 (0.02)		2.29 (0.01)		6.22 (0.03)	
Female	1.30 (0.01)	0.08 (0.07, 0.10)[d]	4.66 (0.02)	0.43 (0.40, 0.46)[d]	2.09 (0.01)	-0.20 (-0.22, -0.18)[d]	6.27 (0.03)	0.05 (0.003, 0.1)[b]
Overall *P*		<0.001		<0.001		<0.001		0.04
Grade								
Secondary 2	1.28 (0.01)	1	4.23 (0.02)	1	2.11 (0.01)	1	6.25 (0.03)	1
Secondary 4	1.28 (0.01)	0 (-0.02, 0.01)	4.52 (0.02)	0.29 (0.27, 0.31)[d]	2.20 (0.01)	0.09 (0.08, 0.11)[d]	6.29 (0.03)	0.04 (0.01, 0.07) [b]
Secondary 6	1.20 (0.01)	-0.08 (-0.10, -0.06)[d]	4.60 (0.02)	0.37 (0.34, 0.4)[d]	2.25 (0.01)	0.14 (0.12, 0.16)[d]	6.21 (0.03)	-0.04 (-0.09, 0.01)
Overall *P*		<0.001		<0.001		<0.001		0.001
Breakfast habits								
Eating at home	1.09 (0.01)	1	4.01 (0.02)	1	1.93 (0.01)	1	5.62 (0.03)	1
Eating away from home	1.25 (0.01)	0.16 (0.14, 0.18)[d]	4.40 (0.02)	0.40 (0.36, 0.44)[d]	2.20 (0.01)	0.27 (0.25, 0.29)[d]	6.29 (0.04)	0.66 (0.60, 0.72)[d]
Skipping	1.42 (0.02)	0.33 (0.30, 0.36)[d]	4.93 (0.03)	0.92 (0.87, 0.97)[d]	2.43 (0.02)	0.50 (0.47, 0.53)[d]	6.84 (0.04)	1.21 (1.14, 1.29)[d]
Overall *P*		<0.001		<0.001		<0.001		<0.001

CI, confidence interval; SE, standard error.

[a] Higher scores indicate the presence of more symptoms of the corresponding problems. Mean (SE and adjusted regression coefficients (βs) (95% CIs) were estimated using generalized estimating equations with the Youth Self-Report scores as continuous outcomes, adjusted for year enrolled in P6, parental educational level and occupation, time-dependent factors in Primary 6 and Secondary 2 and 4, including weight status, and frequency/duration of extracurricular physical activity, and mutually adjusted for each other.

[b] $P<0.05$

[c] $P<0.01$

[d] $P<0.001$
